# Glycolysis Combined with Core Pluripotency Factors to Promote the Formation of Chicken Induced Pluripotent Stem Cells

**DOI:** 10.3390/ani11020425

**Published:** 2021-02-06

**Authors:** Xia Yuan, Chen Zhang, Ruifeng Zhao, Jingyi Jiang, Xiang Shi, Ming Zhang, Hongyan Sun, Qisheng Zuo, Yani Zhang, Jiuzhou Song, Guohong Chen, Bichun Li

**Affiliations:** 1 Key Laboratory of Animal Breeding Reproduction and Molecular Design for Jiangsu Province, College of Animal Science and Technology, Yangzhou University, Yangzhou 225009, China; yuanxia0306@gmail.com (X.Y.); m160647@yzu.edu.cn (C.Z.); zrfnj886@126.com (R.Z.); jiangjy1127@163.com (J.J.); sx19950925@163.com (X.S.); Dat_ming@163.com (M.Z.); hongyans2392@163.com (H.S.); 006664@yzu.edu.cn (Q.Z.); ynzhang@yzu.edu.cn (Y.Z.); yzughc@126.com (G.C.); 2 Joint International Research Laboratory of Agriculture and Agri-Product Safety of Ministry of Education of China, Yangzhou University, Yangzhou 225009, China; 3 Department of Animal & Avian Sciences, University of Maryland, College Park, MD 20742, USA; songj88@umd.edu

**Keywords:** chicken iPSCs, reprogramming, glycolysis, core pluripotency factors

## Abstract

**Simple Summary:**

Chicken embryonic fibroblasts (CEF) can be induced into iPSCs by Oct4, Sox2, Nanog and Lin28 (OSNL). However, the instability of this system leads to low induction efficiency, and the mechanism of reprogramming is still unclear. Therefore, it is vital to study the mechanism of reprogramming and optimize its induction system. In this study, we first note that glycolysis was activated during the reprogramming of CEF using the OSNL induction strategy, and that the further activation of endogenous pluripotent genes promoted the reprogramming process. An optimized OSNL reprogramming cocktail was created by introducing two small-molecule inhibitors (2i-SP), TGF-β inhibitor and MEK/ERK inhibitor, which we termed the “glycolysis activator”, which improved the efficiency of iPSC formation. This study provides an enhanced theoretical basis and suggests further areas for avian cell and embryo engineering research.

**Abstract:**

Somatic cells can be reprogrammed into induced pluripotent stem cells (iPSCs) in vitro. Previously, a lentivirus induction strategy of introducing Oct4, Sox2, Nanog and Lin28 (OSNL) into the iPSC process has been shown as a possible way to produce chicken iPSCs from chicken embryonic fibroblasts, but the induction efficiency of this method was found to be significantly limiting. In order to help resolve this efficiency obstacle, this study seeks to clarify the associated regulation mechanisms and optimizes the reprogramming strategy of chicken iPSCs. This study showed that glycolysis and the expression of glycolysis-related genes correlate with a more efficient reprogramming process. At the same time, the transcription factors Oct4, Sox2 and Nanog were found to activate the expression of glycolysis-related genes. In addition, we introduced two small-molecule inhibitors (2i-SP) as a “glycolysis activator” together with the OSNL cocktail, and found that this significantly improved the induction efficiency of the iPSC process. As such, the study identifies direct molecular connections between core pluripotency factors and glycolysis during the chicken iPSC induction process and, with its results, provides a theoretical basis and technical support for chicken somatic reprogramming.

## 1. Introduction

In contrast to mammals, birds are oviparous animals with unique embryonic development modes. Because of this, certain cellular techniques cannot be easily applied, including somatic cell nuclear transfer (SCNT), in vitro fertilization and embryo transfer, as well as the cryopreservation of ova and embryos. The migration characteristics of avian primordial germ cells (PGCs) have been shown to be useful for offspring production of avian [1,2]. Through the transplantation of cultured chicken PGCs into chicken embryo blood vessels, it was found that donor PGCs can proliferate in the gonad of the recipient embryo and produce chimeric chickens [3,4], suggesting that the allogeneic transplantation of PGCs may be able to help resolve the issue of preserving avian germplasm resources. However, the number of gonads and PGCs derived in blood is clearly inadequate to reach the necessary amounts of typical intravascular injections, which limited the application of such PGC allogeneic transplantations.

In 2006, Takahashi and Yamanaka [5] first transfected the four factors, Oct4, Sox2, Klf4 and c-Myc (OSKM), into mouse fibroblasts via retroviral vectors, and thereby obtained pluripotent stem cells similar to embryonic stem cells (ESCs), which are called induced pluripotent stem cells (iPSCs). If such iPSCs derived from somatic cells were further induced into PGCs, this would undoubtedly be an optimal way to obtain PGCs. However, the corresponding research of somatic cell reprogramming in avian species has remained relatively limited. In 2012, Lu et al. [6] used Nanog, Oct4, Sox2, Klf4, c-Myc and Lin28 (OSKMNL) lentiviral overexpression vectors for the first time to reprogram somatic cells from quails into iPSCs. Meanwhile, previous research by our lab has shown that chicken embryonic fibroblasts (CEF) can be successfully reprogrammed into iPSCs through an induction strategy using Oct4, Sox2, Nanog and Lin28 (OSNL) [7]. However, there are several salient issues that limit the technology’s widespread application, including system instability, low induction efficiency and a lack of clarity of the reprogramming mechanisms. Therefore, it is vital to study the mechanism of reprogramming and optimize its induction system. Some studies have shown that the energy conversion from oxidative phosphorylation to glycolysis [8,9] occurs in the early stage of reprogramming, before the activation of the endogenous pluripotent molecular regulatory network [10]. However, the conversion mechanism between glucose metabolism and exogenous pluripotency factors and the endogenous pluripotency decomposition network has not been reported yet. Therefore, this study aims to explore the effects of glucose metabolism and exogenous pluripotency factors on somatic cell-induced reprogramming.

In this study, we first note that glycolysis was activated during the reprogramming of CEF using the OSNL induction strategy and that the further activation of endogenous pluripotent genes promoted the reprogramming process. An optimized OSNL reprogramming cocktail was created by introducing two small-molecule inhibitors (2i-SP), TGF-β inhibitor and MEK/ERK inhibitor, which we termed the “glycolysis activator”, which improved the efficiency of iPSC formation. This study provides an enhanced theoretical basis and suggests further areas for avian cell and embryo engineering research.

## 2. Materials and Methods

### 2.1. Ethics Approval

Animal experiments were approved by the Institutional Animal Care and Use Committee of the Yangzhou University Animal Experiments Ethics Committee (permit number: SYXK [Su] IACUC 2012-0029). All experimental procedures were performed in accordance with the Regulations for the Administration of Affairs Concerning Experimental Animals approved by the State Council of the People’s Republic of China.

### 2.2. Cell Culture

Chicken embryonic fibroblasts (CEF) were isolated according to our lab’s previously reported method [11]. Chicken embryos hatched for 6–10 days were cut up after removing their head, tail, limbs and viscera; digested with trypsin; centrifuged; and cultured with Dulbecco’s Modified Eagle Medium (DMEM) (Hyclone, Logan, UT, USA) containing 10% Fetal Bovine Serum (FBS) (Gibco, Grand Island, NY, USA). The isolation and culture methods of the chicken ESCs also followed that of our previous report [12]. The blastoderm was aseptically separated from the freshly fertilized eggs; the yolk on it was rinsed with PBS; the blastoderm was blown off, centrifuged, and cultured with ESC medium. The chicken ESC medium was composed of 43.5 mL Knockout DMEM (Gibco, Grand Island, NY, USA), 0.1 mmol/L β-mercaptoethanol (Sigma, St. Louis, MO, USA), 0.4% non-essential amino acids (Sigma, St. Louis, MO, USA), 2% chicken serum (Gibco, Grand Island, NY, USA), 5 ng/mL Stem Cell Factor (SCF) (Sigma, St. Louis, MO, USA), 10 ng/mL Fibroblast Growth Factor-basic (bFGF) (Sigma, St. Louis, MO, USA), 1 ng/mL Leukemia Inhibitory Factor (LIF) (Merck Millipore, Burlington, MA, USA) and 0.5% penicillin (Solarbio, Beijing, China).

### 2.3. Generation of Chicken iPSCs from CEF Cultures

Oct4, Sox2, Nanog and Lin28 (OSNL) overexpressing lentiviral vectors (including EGFP markers) were stored in our lab and applied with lentivirus (Genecreate, Wuhan, China). Schematic diagram of Oct4, Sox2, Nanog and Lin28 (OSNL) overexpression vectors framework were shown in Supplementary Materials File S1. When the cell density reached 60%, the CEF was then transfected with the OSNL reprogramming cocktail, which consisted of Oct4, Sox2, Nanog and Lin28 overexpressing lentiviral vectors in a ratio of 1:1:1:1. The multiplicity of infection rate was 10, and the final concentration of polybrene (Santa Cruz Biotechnology, Santa Cruz, CA, USA) was 5 ng/mL. At 24 h after the transfection, the cells were replaced with DMEM containing 10% FBS and cultured for 72 h. The transfection medium was then replaced with the induction culture medium (the ESC medium), and the induction culture continued until iPSC clones appeared. The composition of ESC medium refers to the 2.2 cell culture method.

For the treatment of compounds, the cells were cultured in the chicken ESC medium and treated with 2i-SP (3 µM SB431542, MCE, Monmouth Junction, NJ, USA; 1 µM PD0325901, MCE, Monmouth Junction, NJ, USA), 20 µM VK3 (Macklin, Shanghai, China) and 40 µM DASA-58 (MCE, Monmouth Junction, NJ, USA). The medium was then replaced every 2 days depending on the cell density.

### 2.4. Alkaline Phosphatase Staining

An azo-coupling alkaline phosphatase staining kit (Solarbio, Beijing, China) was used to determine the alkaline phosphatase activity of the chicken iPSCs. An alkaline phosphatase (ALP) fixative was added for 3 min and an ALP incubation solution shielded from light for 15–20 min. A nuclear red or methyl green staining solution was also added to the counterstain for 3–5 min. Then, the samples were observed under an inverted fluorescence microscope.

### 2.5. Immunofluorescence Staining

4% paraformaldehyde solution (Solarbio, Beijing, China) was applied for 30 min. The antibody blocking solution (PBS containing 10% FBS) was added for 2 h. An antibody (SSEA-1, 1:100–1000) (R&D Systems, Minneapolis, MN, USA) was applied, and then the samples were incubated at 4 °C overnight. The SSEA-1 antibody was Human/Mouse SSEA-1 Alexa Fluor^®^ 594-conjugated Antibody. Then, the samples were stained with 5 ng/μL DAPI (Beyotime, Beijing, China) for 10 min and observed under an inverted fluorescence microscope. Cells were stained red by SSEA-1 antibody and counterstained blue by DAPI.

### 2.6. Flow Cytometry Analysis

The chicken iPSCs were collected via trypsinization and blocked with a blocking buffer (PBS containing 10% FBS) at 37 °C for 2 h. They were then incubated with antibodies (SSEA-1, 1:100–1000) (R&D Systems, Minneapolis, MN, USA) at 4 °C overnight. The SSEA-1 antibody was Human/Mouse SSEA-1 Alexa Fluor^®^ 594-conjugated Antibody. The flow cytometry analysis was performed using FACS LSRFortessa (BD Biosciences, Franklin Lakes, NJ, USA) with at least 10,000 events per experiment. The Flowjo VX software package was used for flow cytometry data analysis. First, drag the flow cytometry file into Flowjo VX. Second, set the negative control sample; double-click the negative control sample file; select FSC-A on the horizontal axis; select SSC-A on the vertical axis; and select the circle gate tool to circle the brighter area on the cell. Double-click the circled cell above; another window pops up; select the channel; select APC-A on the horizontal axis; select SSC-A on the vertical axis; select the circle gate tool; and circle an area on the right side of the cell. Finally, apply the gate circled by the negative control sample to the positive sample. The cells that appear in the gate in the window of APC-A/SSC-A are the positive cells.

### 2.7. Glucose Uptake Assay

The 1 × 10^4^ cells/well were inoculated into a 96-well plate with a black transparent bottom. Untreated cells were used as internal controls. Then, 100 μL of 100-μM 2-NBDG (Thermofisher, Waltham, MA, USA) and glucose-free medium (Solarbio, Beijing, China) was added to cover the sample at 37 °C, 5% CO_2_ and shielded from light for 30 min. A fluorescence microplate reader (Tecan, Männedorf, Switzerland) was used to detect fluorescence, ex465 and em540.

### 2.8. Lactate Production Assay

A Lactic Acid Assay Kit (Nanjing Jiancheng Bioengineering Institute, Nanjing, China) was used for the measurement of lactate production. The induction culture medium supernatant was collected for lactate testing according to the manufacturer’s instructions. The absorbance was determined by a microplate spectrophotometer (Infinite M200 Pro, Tecan Austria GmbH, Grödig, Austria). The amount of lactate generation was calculated as follows: lactate generation (mM) = 3 (OD sample − OD blank)/(OD standard − OD blank).

### 2.9. JC-1 Mitochondrial Membrane Potential Detection

The JC-1 kit (Solarbio, Beijing, China) was used to detect the mitochondrial membrane potential of the cells, and the JC-1 staining working solution and JC-1 buffer solution were prepared according to the manufacturer’s instructions. Then, 1 mL (6-well plate) of fresh medium and 1 mL of JC-1 staining working solution were added and incubated for 20 min, washed twice with pre-chilled JC-1 buffer, followed by the addition 2 mL of medium, and observed under an inverted fluorescent microscope.

### 2.10. qRT-PCR

Total RNA was extracted from cells using a TRNZOL reagent (Tiangen, Beijing, China). A cDNA first-strand synthesis kit (Tiangen, Beijing, China) was then used to synthesize cDNA according to the manufacturer’s instructions. Real-time PCR experiments were performed using the SYBR Green Fluorescence Quantification Kit (Tiangen, Beijing, China) according to the manufacturer’s instructions. Chicken β-actin was used as an internal control. Relative gene expression was calculated using the 2^−ΔΔCt^ method. The primer sequence is shown in Table 1; the primers for endogenous pluripotent genes are Oct4 UTR, Sox2 UTR, Nanog UTR and Lin28 UTR.

### 2.11. Dual Luciferase Reporter Assay

The binding motifs of transcription factors OCT4, SOX2 and NANOG were searched on the website (http://jaspar.genereg.net/ (accessed on 30 January 2021)), and the promoter sequences of key genes for glycolysis were searched on NCBI (https://www.ncbi.nlm.nih.gov/ (accessed on 30 January 2021)). After comparing the binding motif of Oct4, Sox2 and Nanog with the promoter sequences of glycolysis key genes, three common target genes, *Hk1*, *Pfkp* and *Ldha*, of the transcription factors OCT4, SOX2 and NANOG were discovered. The wild-type and mutant double luciferase reporter vectors with common binding sites of Oct4, Sox2 and Nanog in the promoter region of *Hk1*, *Pfkp* and *Ldha* were constructed, respectively (Genecreate, Wuhan, China). DF-1 cells (an immortalized chicken embryo fibroblast cell line) were co-transfected with Oct4; Sox2; Nanog overexpression vectors; and *Hk1*, *Pfkp* and *Ldha* dual luciferase reporter vectors. The pRL-TK vector was used as an internal control. Cells were collected 48 h later, and a dual luciferin detection kit (Vazyme, Nanjing, China) was used to detect luciferase activity.

### 2.12. Embryoid Bodies Formation In Vitro

Chicken iPSCs were enzymatically dissociated after several passages, washed with PBS and then plated in a 24-well plate at a density of 10^5^/well, cultured in differentiation medium containing DMEM high glucose medium, 0.1 mmol/L β-mercaptoethanol, 40 ng/mL human recombinant bone morphogenetic protein 4 (BMP4, R&D Systems, Minneapolis, MN, USA), 0.4% non-essential amino acids, 0.5% penicillin, 10% FBS and 2% chicken serum at 37 °C in a 5% CO_2_ humidified incubator. Fresh medium was added every 2 days.

### 2.13. Statistical Analysis

All experiments were performed in triplicate, and data are expressed as the mean ± standard error. Column statistics and ANOVA were performed to verify the significance of the obtained data by using the Graphpad Prism v.07 software package. Differences with *p* < 0.05 were considered statistically significant. This is presented as * (*p* ≤ 0.05), ** (*p* ≤ 0.01).

## 3. Results

### 3.1. Exogenous OSNL Factors Activate Glycolysis

**Figure 1:**
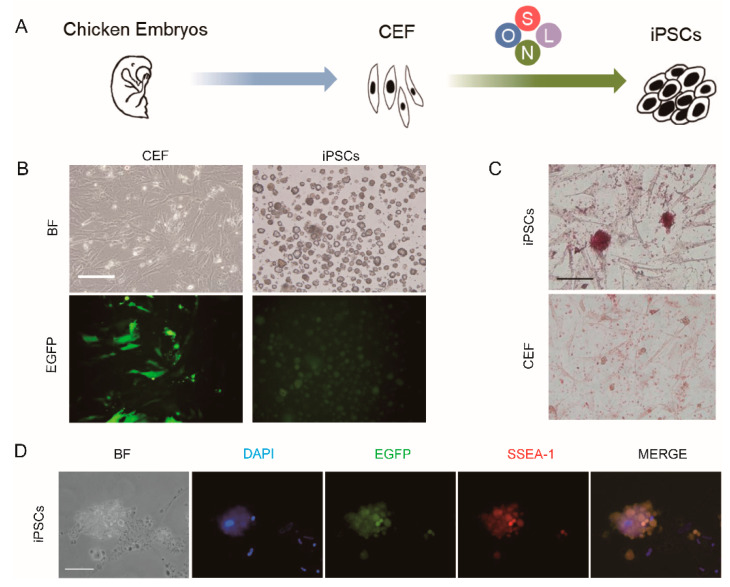
Generation of chicken iPSCs from chicken embryonic fibroblast (CEF) cultures via OSNL (Oct4, Sox2, Nanog, Lin28) induction strategy. (**A**) The strategy of inducing chicken iPSCs from CEF cultures. (**B**) Cell morphology and GFP expression of CEF (taken on the 3rd day of induction of reprogramming) and iPSCs (taken on the 15th day of induction of reprogramming). Scale bars = 100 μm. (**C**) Chicken iPSC clones were stained with an alkaline phosphatase kit. Scale bars = 50 μm. (**D**) Chicken iPSC clones were stained with a mouse monoclonal antibody against SSEA-1. Scale bars = 50 μm.

**Figure 2:**
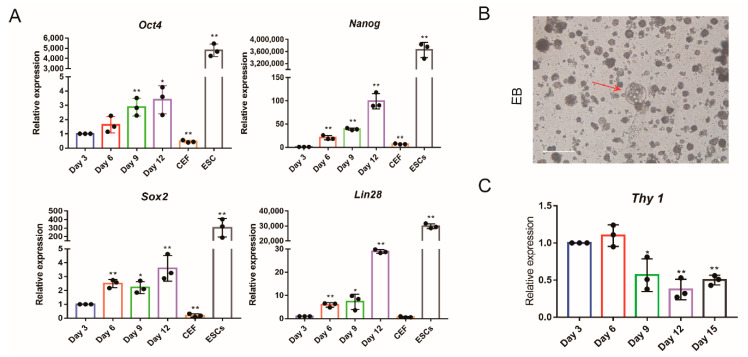
Pluripotency of chicken iPSCs. (**A**) qRT- PCR analysis of expression of endogenous pluripotent genes *Oct4*, *Sox2*, *Nanog* and *Lin28* in CEF transduced with OSNL reprogramming cocktail. (**B**) Embryoid body formation in vitro. Scale bars = 100 μm. (**C**) qRT-PCR analysis of expression of somatic marker gene *Thy1* in CEF transduced with OSNL reprogramming cocktail. * represents *p* < 0.05 and ** represents *p* < 0.01.

In order to obtain chicken iPSCs from somatic cells, we introduced an OSNL cocktail into chicken embryonic fibroblast (Figure 1A). During the 15 day induction period, the morphology of the induced cells became nested ESC clusters (Figure 1B). After the iPSC morphology stabilized, we confirmed the pluripotency of chicken iPSCs by AP (Figure 1C), SSEA-1(Figure 1D), Oct4, Sox2, Nanog expression (Figure 2A) and EBs formation (Figure 2B). qRT-PCR detection revealed that the expression of endogenous pluripotent genes Oct4, Sox2, Nanog and Lin28 gradually increased (Figure 2A). In contrast, the expression of the somatic marker gene Thy-1 gradually decreased (Figure 2C). These results indicated that the chicken embryonic fibroblasts were successfully reprogrammed into iPSCs through the OSNL induction strategy.

**Figure 3:**
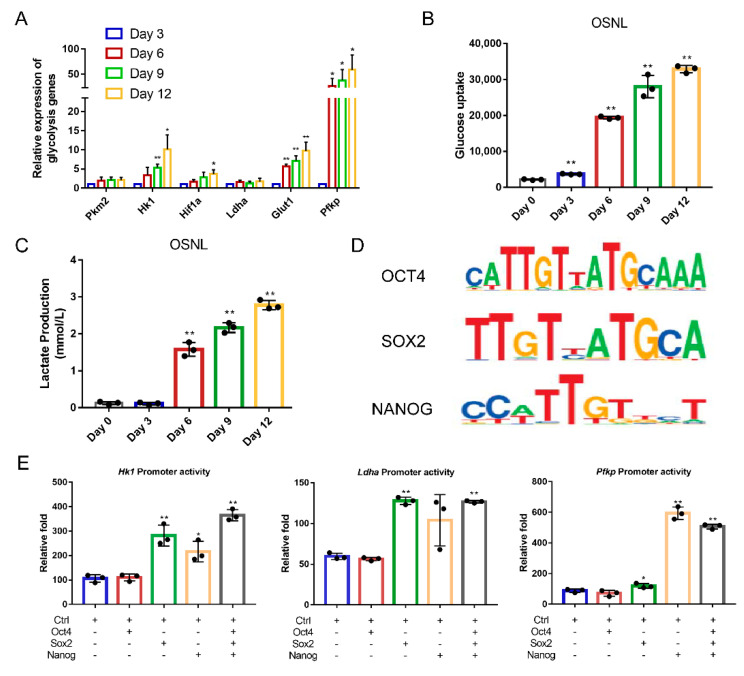
Exogenous OSNL factors activate glycolysis. (**A**) qRT-PCR analysis of expression of glycolysis-related genes in CEF transduced with OSNL reprogramming cocktail. (**B**,**C**) Changes in glucose uptake and lactate production were determined in OSNL reprogramming cocktail transduced CEF. (**D**) Transcription factor binding motif. The binding motifs of transcription factors OCT4, SOX2 and NANOG were searched on the website (http://jaspar.genereg.net/ (accessed on 30 January 2021)), and the promoter sequences of key genes for glycolysis were searched on NCBI (https://www.ncbi.nlm.nih.gov/ (accessed on 30 January 2021)). After comparing the binding motif of Oct4, Sox2 and Nanog with the promoter sequences of glycolysis key genes, three common target genes, *Hk1*, *Pfkp* and *Ldha*, of the transcription factors OCT4, SOX2 and NANOG were discovered. (**E**) Co-transfect Oct4, Sox2 and Nanog overexpression vectors with *Hk1*, *Pfkp* and *Ldha* dual luciferase reporter vectors, respectively, into DF-1 cells. Luciferase activity was detected by a dual luciferin detection kit, normalized to control cells. * represents *p* < 0.05 and ** represents *p* < 0.01.

It is well established that the energy of differentiated cells is primarily from oxidative phosphorylation, while for pluripotent stem cells, it is mainly through glycolysis [13]. As such, the reprogramming of CEF to iPSCs requires the cell’s glucose metabolism to change. Using qRT-PCR detection, we found that the mRNA expression levels of genes encoding enzymes in glycolysis, such as *Hk1*, *Pkm2*, *Pfkp*, *Ldha* and *Glut1*, were significantly upregulated during the reprogramming process (Figure 3A). Examination of glucose uptake and lactate production during reprogramming revealed that they both significantly increased with reprogramming as well (*p* < 0.01) (Figure 3B,C).

In order to study the specific effect on glycolysis regulation by exogenous transcription factors, we conducted a bioinformatics analysis and found that the core pluripotency factors, Oct4, Sox2 and Nanog, have common binding sites in the promoter regions of genes, which encode rate-limiting enzymes in glycolysis, such as *Hk1*, *Pfkp* and *Ldha* (Figure 3D, Supplementary Materials File S2). The dual luciferase reporter assay showed that the overexpression of Sox2 and Nanog significantly increased the transcription activity of *Hk1*, *Pfkp* and *Ldha* genes (*p* < 0.01) (Figure 3E).

### 3.2. Glycolysis Activates the Expression of Endogenous Pluripotent Genes to Promote the Formation of Chicken iPSCs

**Figure 4:**
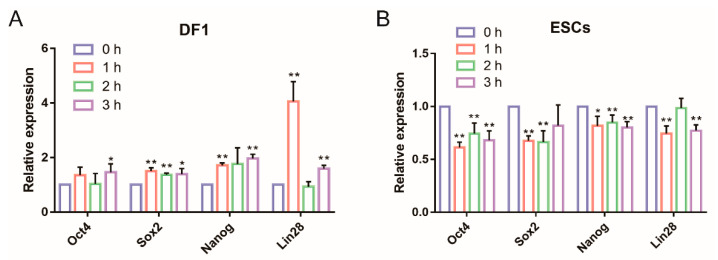
Glycolysis activates the expression of pluripotent genes. (**A**) qRT-PCR analysis of expression of pluripotent genes *Oct4*, *Sox2*, *Nanog* and *Lin28* after adding glycolytic activator DASA-58 to DF-1. (**B**) qRT-PCR analysis of expression of pluripotent genes *Oct4*, *Sox2*, *Nanog* and *Lin28* after adding glycolytic inhibitor VK3 to ESCs. * represents *p* < 0.05 and ** represents *p* < 0.01.

During the reprogramming process, the endogenous pluripotent gene network is gradually activated. In order to determine whether glycolysis can further activate the expression of endogenous pluripotent genes, the glycolytic activator DASA-58 or glycolytic inhibitor VK3 was added to DF-1 cells or ESCs, respectively. Following this treatment, the transient expression of pluripotent genes was tested at hourly intervals for 3 h. The results showed that after adding the glycolytic activator DASA-58 to DF-1, the expression of pluripotent genes *Oct4*, *Sox2*, *Nanog* and *Lin28* was significantly upregulated (Figure 4A). However, the expression of these pluripotent genes was significantly downregulated when the glycolytic inhibitor VK3 was added to ESCs (Figure 4B).

**Figure 5:**
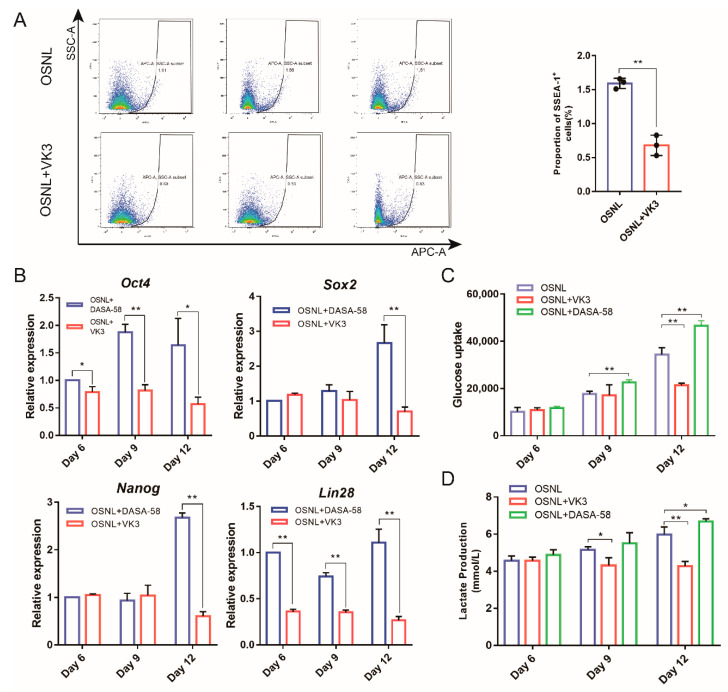
Glycolysis activates the expression of endogenous pluripotent genes to promote the formation of chicken iPSCs. (**A**) The proportion of SSEA-1 positive cells after adding glycolytic inhibitor VK3 detected by flow cytometry. (**B**) qRT-PCR analysis of expression of pluripotent genes *Oct4*, *Sox2*, *Nanog* and *Lin28* after adding glycolytic activator DASA-58 or glycolytic inhibitor VK3. (**C**) Changes in glucose uptake after adding glycolytic activator DASA-58 or inhibitor VK3. (**D**) Changes in lactate production after adding glycolytic activator DASA-58 or inhibitor VK3. * represents *p* < 0.05 and ** represents *p* < 0.01.

In order to study the effect of glycolysis on iPSC induction, we also performed flow cytometry analysis on the 15th day of induction. The results showed that the proportion of SSEA-1 positive cells decreased significantly (*p* < 0.01) when the glycolytic inhibitor VK3 was added (Figure 5A). Additionally, glucose uptake; lactate production; and the expression of pluripotent genes *Oct4*, *Sox2*, *Nanog* and *Lin28* were downregulated (*p* < 0.01) (Figure 5B–D). However, when the glycolytic activator DASA-58 was added, glucose uptake; lactate production; and the expression of *Oct4*, *Sox2*, *Nanog* and *Lin28* were significantly upregulated (*p* < 0.05) (Figure 5B–D).

### 3.3. 2i-SP Increases Glycolysis Levels to Promote Chicken iPSC Formation

**Figure 6:**
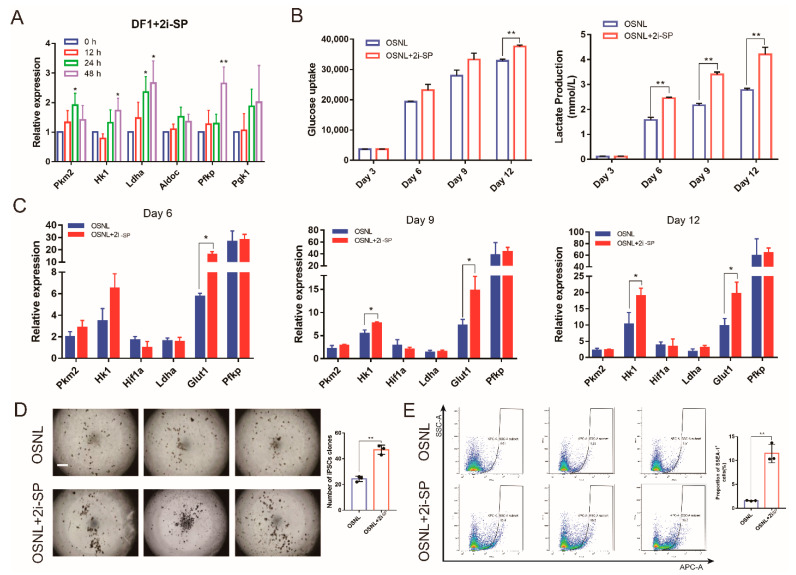
2i-SP increases glycolysis level to promote chicken iPSC formation. (**A**) qRT-PCR analysis of expression of genes encoding related enzymes in glycolysis after adding 2i-SP (SB431542, PD0325901) to DF-1. (**B**) Changes in glucose uptake and lactate production after adding 2i-SP. (**C**) qRT-PCR analysis of expression of genes encoding related enzymes in glycolysis after adding 2i-SP. (**D**) Changes of cell morphology and the number of chicken iPSC clones after adding 2i-SP. Scale bars = 100 μm. (**E**) The proportion of SSEA-1 positive cells after adding 2i-SP was detected by flow cytometry. * Represents *p* < 0.05 and ** represents *p* < 0.01.

In order to improve the induction efficiency of iPSCs, two small-molecule inhibitors (2i-SP; SB431542, a TGF-β inhibitor; and PD0325901, a MEK/ERK inhibitor) were introduced. 2i-SP can activate glycolysis, and it was introduced to optimize the induction system of iPSCs of mice [14,15,16]. These compounds are widely used in embryonic stem cell cultures and iPSC induction, but their effectiveness in non-mammal species remains unproven. In order to ensure their basic function effect on chicken cells, we introduced these compounds into the DF-1 culture medium. We added 2i-SP to DF-1 to be cultured and collected cells at twelve hour intervals for the subsequent 48 h. qRT-PCR detection showed that the expression of glycolysis-related genes *Pkm2*, *Ldha*, *Pfkp* and *Hk1* was significantly upregulated after adding 2i-SP (*p* < 0.05) (Figure 6A).

Then, we added 2i-SP to the OSNL reprogramming cocktail to induce CEF reprogramming. qRT-PCR showed that the mRNA expressions of glycolysis-related genes *Hk1* and *Glut1* were all upregulated after adding 2i-SP (Figure 6C), and glucose uptake and lactate production also increased in varying degrees (Figure 6B). At the same time, the number of iPSC clones induced after adding 2i-SP significantly increased (*p* < 0.01) (Figure 6D). Flow cytometry showed that the proportion of SSEA-1 positive cells also increased significantly (*p* < 0.01) (Figure 6E).

### 3.4. 2i-SP Inhibits Oxidative Phosphorylation to Promote Chicken iPSC Formation

**Figure 7:**
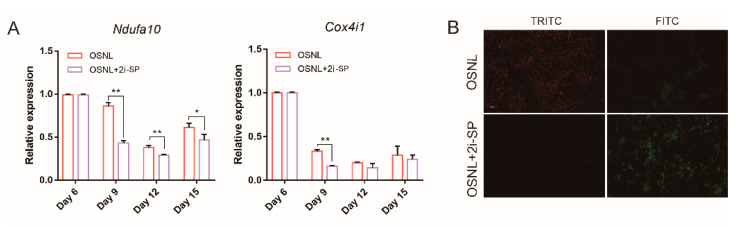
2i-SP inhibits oxidative phosphorylation to promote chicken iPSC formation. (**A**) qRT-PCR analysis of expression of genes encoding related enzymes in oxidative phosphorylation after adding 2i-SP. (**B**) Changes in cell mitochondrial membrane potential after adding 2i-SP detected by the JC-1 kit. When the mitochondrial membrane potential is high, JC-1 can form polymers and produce red fluorescence (~590 nm, TRITC). On the contrary, when the mitochondrial membrane potential is low, JC-1 is a monomer and produces green fluorescence (~525 nm; FITC). Scale bars = 100 μm. * Represents *p* < 0.05 and ** represents *p* < 0.01.

It has been shown that oxidative phosphorylation levels will change with the variation of glycolysis in the somatic reprogramming process [10]. To find out whether 2i-SP can regulate oxidative phosphorylation as well, we used qRT-PCR analysis to detect that the expression levels of *Ndufa10* and *Cox4i1*, which encode related enzymes in oxidative phosphorylation. These genes were inhibited after adding 2i-SP to the OSNL reprogramming cocktail (Figure 7A), and the JC-1 mitochondrial membrane potential measurement showed that mitochondrial activity decreased significantly (Figure 7B).

## 4. Discussion

**Figure 8:**
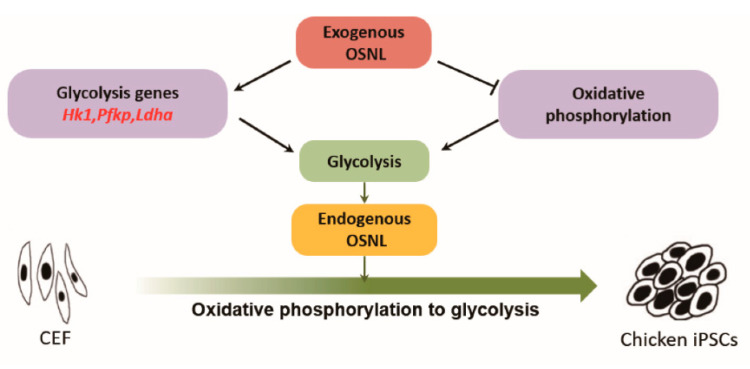
Schematic of function of 2i-SP during CEF reprogramming to chicken iPSCs.

In this study, we found that during the reprogramming of CEF to iPSCs via an OSNL induction strategy, exogenous core pluripotency factors activated glycolysis and further activated the endogenous pluripotent gene network to promote iPSC formation (Figure 8). Additionally, the “glycolytic activator” 2i-SP was used to attempt to optimize the induction system of chicken iPSCs, and the formation efficiency of chicken iPSCs was increased from 1.59% to 11.47%.

The process of somatic reprogramming is regulated by many factors. Silva [17] originally discovered through a cell fusion study that the reactivation of pluripotent genes is the key factor to reprogramming. Oct4 [18], Sox2 [19], c-Myc [20], Klf4 [21], Nanog [22] and Lin28 [23] are all critical regulators to maintain pluripotency and self-renewal properties in pluripotent stem cells. In this study, we successfully obtained chicken iPSCs from somatic cells by an OSNL induction strategy. iPSCs can be subcultured stably for a long time in vitro; they are ideal seed cells in transgenic technology. They are used as gene targeting recipient cells and have broad application prospects in the production of transgenic animals. Especially for species that have not established ES cell lines to date, iPSC cells may become their ES cell replacement for scientific research and production practice. In poultry, iPSCs can be used to induce PGCs [24] and further in the production of transgenic poultry and the conservation of rare birds.

In this study, we found that the addition of exogenous core pluripotency factors caused the expression of endogenous glycolysis-related genes, suggesting that the reprogramming process was jointly regulated by intracellular and extracellular signals. Kim et al. [25] found that the core pluripotency factors can directly regulate glycolysis by controlling the expression of glycolytic enzymes when studying the metabolic characteristics of embryonic stem cells. From our work, we found that the core pluripotency factors activate the expression of glycolysis-related genes, and the activated glycolysis can further promote the expression of endogenous pluripotent genes. Therefore, glycolysis seems to be a primary axis connecting intracellular and extracellular signals.

The process of somatic reprogramming involves not only the activation of pluripotent genes but also the reprogramming of cellular metabolism. Liu [26] discovered that energy metabolism plays a critical role in reprogramming. In this study, we found that glycolysis was activated during CEF reprogramming, which was induced by exogenous OSNL factors, indicating that glycolysis is involved in the iPSC reprogramming process. Moreover, we found that the transcription of key genes in glycolysis was regulated by Sox2 and Nanog. Meanwhile, glycolysis can increase the expression of endogenous pluripotent genes and promote the formation of chicken iPSCs through feedback regulation. However, in Liu’s [26] research, the overexpression of the glycolytic genes *Pdk1* and *Pgam2* did not promote the formation of iPSCs. This result may be due to the fact that *Pdk1* and *Pgam2* are not key rate-limiting enzymes for glycolysis [27].

During the reprogramming process, the formation of iPSCs may be limited in multiple ways. In order to help resolve induction efficiency issues, researchers have tried to promote the formation of iPSCs by adding small-molecule compounds. Esteban [28] found that vitamin C can promote the formation of mouse and human iPSCs, while Huangfu [29] increased the induction efficiency of human iPSCs using valproic acid. In this study, based on our finding that glycolysis can promote the induction of chicken iPSCs, we searched for suitable small-molecule compounds. 2i-SP has been reported as being able to promote glycolysis by inhibiting the activities of MEK/ERK and TGF-β and relieving the phosphorylation of GSK3-β [14,15,16]. In addition, various studies have shown that 2i-SP can improve the formation efficiency of mouse and human iPSCs [30,31,32]. For these reasons, we chose to use 2i-SP to optimize the study’s OSNL induction strategy. Furthermore, we found that the level of oxidative phosphorylation decreased after adding 2i-SP in the reprogramming process of CEF to chicken iPSCs. It has been reported that the overexpression of oxidative phosphorylation gene PGC-1 α can inhibit the formation of iPSCs [26], indicating that oxidative phosphorylation was involved in reprogramming. Based on these initial results, we believe the feedback regulation role of glycolysis in oxidative phosphorylation in the formation of chicken iPSCs should be further studied.

## 5. Conclusions

In summary, exogenous transcription factor OSN activated glycolysis and further stimulated the endogenous pluripotent gene expression network in the process of reprogramming CEF to iPSCs by OSNL (Oct4/Sox2/Nanog/Lin28) reprogramming cocktail, which promoted the formation of chicken iPSCs. The small molecule compound 2i-SP (TGF-β inhibitor and MEK/ERK inhibitor) activates glycolysis and significantly improves the induction efficiency of chicken iPSCs. These results increase our understanding of the mechanism of reprogramming, provide new insights into metabolic changes and optimization of the reprogramming induction system and provide a broader theoretical basis and technical support for somatic induced reprogramming.

## Figures and Tables

**Table 1:** Primers for qRT-PCR.

Gene	Forward Primers (5′–3′)	Reverse Primers (5′–3′)
Oct4 UTR	CGGGATCTCCATGAACAACAG	CTGGCCCCAGGCAGGTAA
Sox2 UTR	CATATGTAAGACAAAGGGG	AAGGTCCAGAATTTCTAATAA
Nanog UTR	GTATGCAACCAGCTCACC	TAGTAGTGTCCGCACCTAAC
Lin28 UTR	AAAGCCAATGCCAAGTGA	CAAACAAACCCAAAGATACG
Oct4	TGCAATGCAGAGCAAGTGCTGG	ACTGGGCTTCACACATTTGCGG
Sox2	ACTCGGCCGCGAACAACCAG	GCCCCGAGCCGTTTGCTGAT
Nanog	TGCACACCAGGCTTACAGCAGTG	TGCTGGGTGTTGCAGCTTGTTC
Lin28	ACACCCGTCTGGGCAACGAC	CCCGCTGGATGCGCTTCATC
Thy1	GACTGCCGCTATGAGAACA	GGAGACGCTGATGGTGCT
Hk1	CCTCTTGGCTTCACATTC	TTCACAGTTTGGGTCTTCAT
Pkm2	GGCACCCACGAGTATCAT	CATTGTCCAGCGTCACTTT
Pfkp	GCCACAACAAACCTATAACA	ATCAAAGGCAGACGAACA
Ldha	TGGGCATCCATCCTCTGA	CCTGCTTGTGAACCTCCT
Glut1	TGTTTGGCTTGGACTTGAT	TCTTGAGGACGCTCTTGG
Hif1a	TTGACAAGGCATCCATTA	TCCTCAGAAAGCACCATA
Aldoc	CTGACGACGGCACTCCTTT	GACAGCCCATCCAGACCCT
Pgk1	AGGGCTGCATCACCATTA	CCACCTCCAGTGCTAACG
Ndufa10	TGAGCGAGACCAGTGAAA	GGAATGAACCGAGGGATA
Cox4i1	TTTCAGCCATCCAGCATA	GAAGAGCACTCCACCAAG
β-actin	CAGCCATCTTTCTTGGGTAT	CTGTGATCTCCTTCTGCATCC

## Data Availability

The data presented in this study are available on request from the corresponding author.

## Supplementary Materials

The following are available online at https://www.mdpi.com/2076-2615/11/2/425/s1. File S1: Schematic diagram of Oct4, Sox2, Nanog and Lin28 (OSNL) overexpression vectors framework. File S2: OSN (OCT4, SOX2, NANOG) binding sites on the flanking DNA region of the glycolysis gene promoter.
