# Supplementary material for: Glycolysis Combined with Core Pluripotency Factors to Promote the Formation of Chicken Induced Pluripotent Stem Cells
Source: Animals (Basel). 2021 Feb 6;11(2):425. doi: 10.3390/ani11020425 (PMC7915628; doi:10.3390/ani11020425)
Supplement: Supplementary file 1 [file animals-11-00425-s001.zip › animals-1076207-suppl-final/Supplemental Materials/Supplemental Material 1-Schematic diagram of Oct4, Sox2, Nanog and Lin28 (OSNL) overexpression vectors framework.docx]

**Supplemental Material S1**

**Schematic diagram of Oct4, Sox2, Nanog and Lin28 (OSNL) carrier framework**


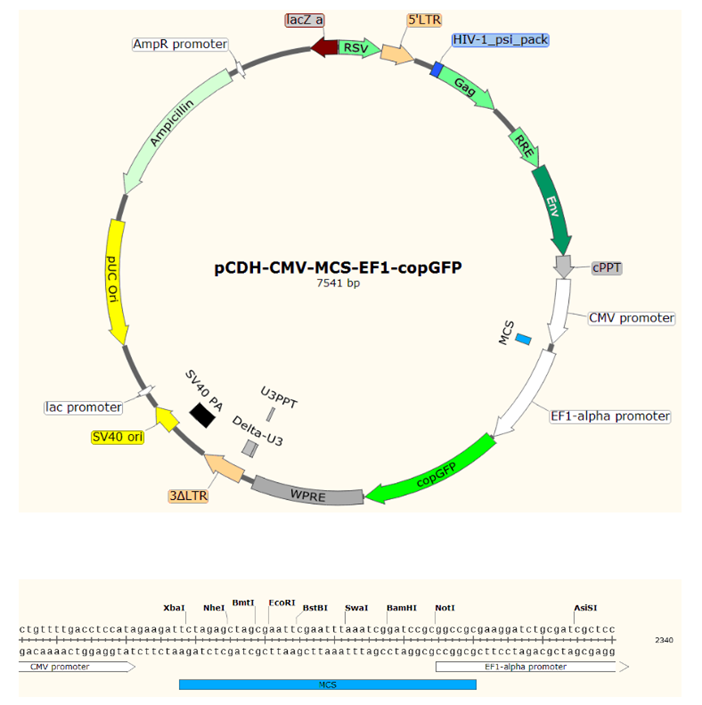


LOCUS pCDH-CMV-MCS-EF1-copGFP 7541 bp ds-DNA circular SYN 25-JUL-2016

DEFINITION pCDH-CMV-MCS-EF1-copGFP.

SOURCE http://www.biofeng.com

ORGANISM synthetic DNA construct

REFERENCE 1 (bases 1 to 7541)

ORIGIN

1 acgcgtgtag tcttatgcaa tactcttgta gtcttgcaac atggtaacga tgagttagca

61 acatgcctta caaggagaga aaaagcaccg tgcatgccga ttggtggaag taaggtggta

121 cgatcgtgcc ttattaggaa ggcaacagac gggtctgaca tggattggac gaaccactga

181 attgccgcat tgcagagata ttgtatttaa gtgcctagct cgatacaata aacgggtctc

241 tctggttaga ccagatctga gcctgggagc tctctggcta actagggaac ccactgctta

301 agcctcaata aagcttgcct tgagtgcttc aagtagtgtg tgcccgtctg ttgtgtgact

361 ctggtaacta gagatccctc agaccctttt agtcagtgtg gaaaatctct agcagtggcg

421 cccgaacagg gacctgaaag cgaaagggaa accagagctc tctcgacgca ggactcggct

481 tgctgaagcg cgcacggcaa gaggcgaggg gcggcgactg gtgagtacgc caaaaatttt

541 gactagcgga ggctagaagg agagagatgg gtgcgagagc gtcagtatta agcgggggag

601 aattagatcg cgatgggaaa aaattcggtt aaggccaggg ggaaagaaaa aatataaatt

661 aaaacatata gtatgggcaa gcagggagct agaacgattc gcagttaatc ctggcctgtt

721 agaaacatca gaaggctgta gacaaatact gggacagcta caaccatccc ttcagacagg

781 atcagaagaa cttagatcat tatataatac agtagcaacc ctctattgtg tgcatcaaag

841 gatagagata aaagacacca aggaagcttt agacaagata gaggaagagc aaaacaaaag

901 taagaccacc gcacagcaag cggccactga tcttcagacc tggaggagga gatatgaggg

961 acaattggag aagtgaatta tataaatata aagtagtaaa aattgaacca ttaggagtag

1021 cacccaccaa ggcaaagaga agagtggtgc agagagaaaa aagagcagtg ggaataggag

1081 ctttgttcct tgggttcttg ggagcagcag gaagcactat gggcgcagcc tcaatgacgc

1141 tgacggtaca ggccagacaa ttattgtctg gtatagtgca gcagcagaac aatttgctga

1201 gggctattga ggcgcaacag catctgttgc aactcacagt ctggggcatc aagcagctcc

1261 aggcaagaat cctggctgtg gaaagatacc taaaggatca acagctcctg gggatttggg

1321 gttgctctgg aaaactcatt tgcaccactg ctgtgccttg gaatgctagt tggagtaata

1381 aatctctgga acagattgga atcacacgac ctggatggag tgggacagag aaattaacaa

1441 ttacacaagc ttaatacact ccttaattga agaatcgcaa aaccagcaag aaaagaatga

1501 acaagaatta ttggaattag ataaatgggc aagtttgtgg aattggttta acataacaaa

1561 ttggctgtgg tatataaaat tattcataat gatagtagga ggcttggtag gtttaagaat

1621 agtttttgct gtactttcta tagtgaatag agttaggcag ggatattcac cattatcgtt

1681 tcagacccac ctcccaaccc cgaggggacc cgacaggccc gaaggaatag aagaagaagg

1741 tggagagaga gacagagaca gatccattcg attagtgaac ggatctcgac ggtatcggtt

1801 aacttttaaa agaaaagggg ggattggggg gtacagtgca ggggaaagaa tagtagacat

1861 aatagcaaca gacatacaaa ctaaagaatt acaaaaacaa attacaaaat tcaaaatttt

1921 atcgatacta gtattatgcc cagtacatga ccttatggga ctttcctact tggcagtaca

1981 tctacgtatt agtcatcgct attaccatgg tgatgcggtt ttggcagtac atcaatgggc

2041 gtggatagcg gtttgactca cggggatttc caagtctcca ccccattgac gtcaatggga

2101 gtttgttttg gcaccaaaat caacgggact ttccaaaatg tcgtaacaac tccgccccat

2161 tgacgcaaat gggcggtagg cgtgtacggt gggaggttta tataagcaga gctcgtttag

2221 tgaaccgtca gatcgcctgg agacgccatc cacgctgttt tgacctccat agaagattct

2281 agagctagcg aattcgaatt taaatcggat ccgcggccgc gaaggatctg cgatcgctcc

2341 ggtgcccgtc agtgggcaga gcgcacatcg cccacagtcc ccgagaagtt ggggggaggg

2401 gtcggcaatt gaacgggtgc ctagagaagg tggcgcgggg taaactggga aagtgatgtc

2461 gtgtactggc tccgcctttt tcccgagggt gggggagaac cgtatataag tgcagtagtc

2521 gccgtgaacg ttctttttcg caacgggttt gccgccagaa cacagctgaa gcttcgaggg

2581 gctcgcatct ctccttcacg cgcccgccgc cctacctgag gccgccatcc acgccggttg

2641 agtcgcgttc tgccgcctcc cgcctgtggt gcctcctgaa ctgcgtccgc cgtctaggta

2701 agtttaaagc tcaggtcgag accgggcctt tgtccggcgc tcccttggag cctacctaga

2761 ctcagccggc tctccacgct ttgcctgacc ctgcttgctc aactctacgt ctttgtttcg

2821 ttttctgttc tgcgccgtta cagatccaag ctgtgaccgg cgcctacgct agatggagag

2881 cgacgagagc ggcctgcccg ccatggagat cgagtgccgc atcaccggca ccctgaacgg

2941 cgtggagttc gagctggtgg gcggcggaga gggcaccccc aagcagggcc gcatgaccaa

3001 caagatgaag agcaccaaag gcgccctgac cttcagcccc tacctgctga gccacgtgat

3061 gggctacggc ttctaccact tcggcaccta ccccagcggc tacgagaacc ccttcctgca

3121 cgccatcaac aacggcggct acaccaacac ccgcatcgag aagtacgagg acggcggcgt

3181 gctgcacgtg agcttcagct accgctacga ggccggccgc gtgatcggcg acttcaaggt

3241 ggtgggcacc ggcttccccg aggacagcgt gatcttcacc gacaagatca tccgcagcaa

3301 cgccaccgtg gagcacctgc accccatggg cgataacgtg ctggtgggca gcttcgcccg

3361 caccttcagc ctgcgcgacg gcggctacta cagcttcgtg gtggacagcc acatgcactt

3421 caagagcgcc atccacccca gcatcctgca gaacgggggc cccatgttcg ccttccgccg

3481 cgtggaggag ctgcacagca acaccgagct gggcatcgtg gagtaccagc acgccttcaa

3541 gacccccatc gccttcgcca gatcccgcgc tcagtcgtcc aattctgccg tggacggcac

3601 cgccggaccc ggctccaccg gatctcgctg tcgacaatca acctctggat tacaaaattt

3661 gtgaaagatt gactggtatt cttaactatg ttgctccttt tacgctatgt ggatacgctg

3721 ctttaatgcc tttgtatcat gctattgctt cccgtatggc tttcattttc tcctccttgt

3781 ataaatcctg gttgctgtct ctttatgagg agttgtggcc cgttgtcagg caacgtggcg

3841 tggtgtgcac tgtgtttgct gacgcaaccc ccactggttg gggcattgcc accacctgtc

3901 agctcctttc cgggactttc gctttccccc tccctattgc cacggcggaa ctcatcgccg

3961 cctgccttgc ccgctgctgg acaggggctc ggctgttggg cactgacaat tccgtggtgt

4021 tgtcggggaa atcatcgtcc tttccttggc tgctcgcctg tgttgccacc tggattctgc

4081 gcgggacgtc cttctgctac gtcccttcgg ccctcaatcc agcggacctt ccttcccgcg

4141 gcctgctgcc ggctctgcgg cctcttccgc gtcttcgcct tcgccctcag acgagtcgga

4201 tctccctttg ggccgcctcc ccgcctggta cctttaagac caatgactta caaggcagct

4261 gtagatctta gccacttttt aaaagaaaag gggggactgg aagggctaat tcactcccaa

4321 cgaaaataag atctgctttt tgcttgtact gggtctctct ggttagacca gatctgagcc

4381 tgggagctct ctggctaact agggaaccca ctgcttaagc ctcaataaag cttgccttga

4441 gtgcttcaag tagtgtgtgc ccgtctgttg tgtgactctg gtaactagag atccctcaga

4501 cccttttagt cagtgtggaa aatctctagc agtagtagtt catgtcatct tattattcag

4561 tatttataac ttgcaaagaa atgaatatca gagagtgaga ggaacttgtt tattgcagct

4621 tataatggtt acaaataaag caatagcatc acaaatttca caaataaagc atttttttca

4681 ctgcattcta gttgtggttt gtccaaactc atcaatgtat cttatcatgt ctggctctag

4741 ctatcccgcc cctaactccg cccagttccg cccattctcc gccccatggc tgactaattt

4801 tttttattta tgcagaggcc gaggccgcct cggcctctga gctattccag aagtagtgag

4861 gaggcttttt tggaggccta gacttttgca gagacggccc aaattcgtaa tcatggtcat

4921 agctgtttcc tgtgtgaaat tgttatccgc tcacaattcc acacaacata cgagccggaa

4981 gcataaagtg taaagcctgg ggtgcctaat gagtgagcta actcacatta attgcgttgc

5041 gctcactgcc cgctttccag tcgggaaacc tgtcgtgcca gctgcattaa tgaatcggcc

5101 aacgcgcggg gagaggcggt ttgcgtattg ggcgctcttc cgcttcctcg ctcactgact

5161 cgctgcgctc ggtcgttcgg ctgcggcgag cggtatcagc tcactcaaag gcggtaatac

5221 ggttatccac agaatcaggg gataacgcag gaaagaacat gtgagcaaaa ggccagcaaa

5281 aggccaggaa ccgtaaaaag gccgcgttgc tggcgttttt ccataggctc cgcccccctg

5341 acgagcatca caaaaatcga cgctcaagtc agaggtggcg aaacccgaca ggactataaa

5401 gataccaggc gtttccccct ggaagctccc tcgtgcgctc tcctgttccg accctgccgc

5461 ttaccggata cctgtccgcc tttctccctt cgggaagcgt ggcgctttct catagctcac

5521 gctgtaggta tctcagttcg gtgtaggtcg ttcgctccaa gctgggctgt gtgcacgaac

5581 cccccgttca gcccgaccgc tgcgccttat ccggtaacta tcgtcttgag tccaacccgg

5641 taagacacga cttatcgcca ctggcagcag ccactggtaa caggattagc agagcgaggt

5701 atgtaggcgg tgctacagag ttcttgaagt ggtggcctaa ctacggctac actagaagga

5761 cagtatttgg tatctgcgct ctgctgaagc cagttacctt cggaaaaaga gttggtagct

5821 cttgatccgg caaacaaacc accgctggta gcggtggttt ttttgtttgc aagcagcaga

5881 ttacgcgcag aaaaaaagga tctcaagaag atcctttgat cttttctacg gggtctgacg

5941 ctcagtggaa cgaaaactca cgttaaggga ttttggtcat gagattatca aaaaggatct

6001 tcacctagat ccttttaaat taaaaatgaa gttttaaatc aatctaaagt atatatgagt

6061 aaacttggtc tgacagttac caatgcttaa tcagtgaggc acctatctca gcgatctgtc

6121 tatttcgttc atccatagtt gcctgactcc ccgtcgtgta gataactacg atacgggagg

6181 gcttaccatc tggccccagt gctgcaatga taccgcgaga cccacgctca ccggctccag

6241 atttatcagc aataaaccag ccagccggaa gggccgagcg cagaagtggt cctgcaactt

6301 tatccgcctc catccagtct attaattgtt gccgggaagc tagagtaagt agttcgccag

6361 ttaatagttt gcgcaacgtt gttgccattg ctacaggcat cgtggtgtca cgctcgtcgt

6421 ttggtatggc ttcattcagc tccggttccc aacgatcaag gcgagttaca tgatccccca

6481 tgttgtgcaa aaaagcggtt agctccttcg gtcctccgat cgttgtcaga agtaagttgg

6541 ccgcagtgtt atcactcatg gttatggcag cactgcataa ttctcttact gtcatgccat

6601 ccgtaagatg cttttctgtg actggtgagt actcaaccaa gtcattctga gaatagtgta

6661 tgcggcgacc gagttgctct tgcccggcgt caatacggga taataccgcg ccacatagca

6721 gaactttaaa agtgctcatc attggaaaac gttcttcggg gcgaaaactc tcaaggatct

6781 taccgctgtt gagatccagt tcgatgtaac ccactcgtgc acccaactga tcttcagcat

6841 cttttacttt caccagcgtt tctgggtgag caaaaacagg aaggcaaaat gccgcaaaaa

6901 agggaataag ggcgacacgg aaatgttgaa tactcatact cttccttttt caatattatt

6961 gaagcattta tcagggttat tgtctcatga gcggatacat atttgaatgt atttagaaaa

7021 ataaacaaat aggggttccg cgcacatttc cccgaaaagt gccacctgac gtctaagaaa

7081 ccattattat catgacatta acctataaaa ataggcgtat cacgaggccc tttcgtctcg

7141 cgcgtttcgg tgatgacggt gaaaacctct gacacatgca gctcccggag acggtcacag

7201 cttgtctgta agcggatgcc gggagcagac aagcccgtca gggcgcgtca gcgggtgttg

7261 gcgggtgtcg gggctggctt aactatgcgg catcagagca gattgtactg agagtgcacc

7321 atatgcggtg tgaaataccg cacagatgcg taaggagaaa ataccgcatc aggcgccatt

7381 cgccattcag gctgcgcaac tgttgggaag ggcgatcggt gcgggcctct tcgctattac

7441 gccagctggc gaaaggggga tgtgctgcaa ggcgattaag ttgggtaacg ccagggtttt

7501 cccagtcacg acgttgtaaa acgacggcca gtgccaagct g


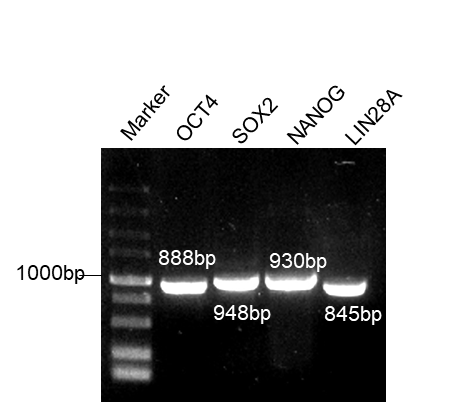


Figure 1. Electrophoretic map of Oct4, Sox2, Nanog, Lin28A PCR amplification.


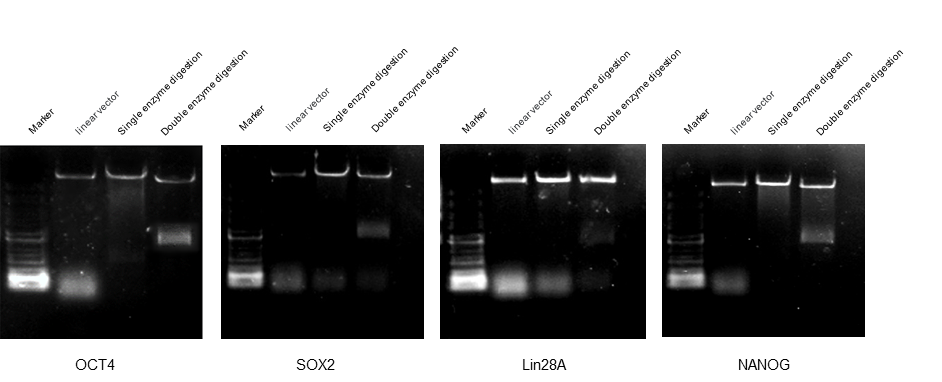


Figure 2. Identification of recombinant vector by restriction enzyme digestion.
