# Supplementary material for: Glycolysis Combined with Core Pluripotency Factors to Promote the Formation of Chicken Induced Pluripotent Stem Cells
Source: Animals (Basel). 2021 Feb 6;11(2):425. doi: 10.3390/ani11020425 (PMC7915628; doi:10.3390/ani11020425)
Supplement: Supplementary file 1 [file animals-11-00425-s001.zip › animals-1076207-suppl-final/Supplemental Materials/Supplemental Material 2-OCT4, SOX2, NANOG (OSN) binding sites on the flanking DNA region of glycolysis gene promoter.docx]

**Supplemental Material S2**

**OCT4, SOX2, NANOG (OSN) binding sites on the flanking DNA region of glycolysis gene promoter**


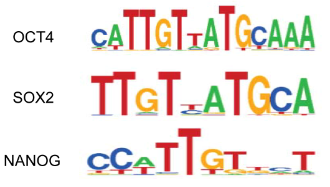


**1. The flanking DNA region of *Hk1* gene**

>NC_006093.5:11574962-11576962 Gallus gallus breed Red Jungle Fowl isolate RJF #256 chromosome 6, GRCg6a

TACCAGAAGTTTAATTAATGTTTTCTCCTCTCTTGTTGATCCCAGTATGCTGTAGGTGCACATTGAAAGTGCTGTAAAGATGATGGTCATGTGTATCTGAGAGAGAAGTGCCAGTGTATTTTGATTTTGTCCTGGGAGTGGGTGGGCAGGAGGAAGACGATCTGTTTCACTGCCACCATGTCTTCTTTTCTCCTTTTTGTTGGGAAGGATAAAAGCTGTTTGGAGAAGAAGGGTAGGATATGAGAGGAGTATGGCACATCAGCAGTCAGAGCTTCGCATAGATTTGTTACTGCAGATTGGAGCAACTTCAAGTCACAGTAATGGGGGGAAAAGGTAACAAATACAAGATTGTGATGTGTTACAAGCAGCAAATTGCAAGGACAACTAAGGGAGGTAACTAAGTCTTTTGGCTCAAATTTGTATTACTTTGTGAGCAGGATAAAAGGGGAAGGAGGAGCAGGGTGGCTGCTGTACCAGAACCCATCTCCTTTGCACTCAGAGGTGTGAGGTTGCACCCAGCCTTGGGCATATACAAAATACCAGGGTTCAGCTCTCAGAACACTGGTGCTGTTGATTCAGTGCAATATTTTTGTGCCTGCTATATGGAACTTTGGCTCGTTAAAGGTTTCTGTGCACCTGGAAAGGCCCTGGGAAGTCCCCTTTCCTATAGGGAAACCATATGGTCATGATGCTGGGCCATCTGTTTAGCTGTGGGACCAGGAGAACTCCCATGAGGCTGCTGGAGAGGATTGTCCTCCTCACTGCCAGCTGTGGCCCCCTGGGAGCCACAGAGGCTGTCCTGGGCTGGCACAGAAAGCTGGGAGGCACTCACTGCCTCGACAATGGGGGAAAGGCACCTTAGGGCTGAGCCAAGGCCGCTCTGGCTCTGGATGCATGCCAACATACATAGAGCATGGTTAGGATGGCGAGAATGGTTGTTGGGGCACAAGCGGTTAATGGAGCAGATCAGCTGCTGATGGACAACTGTGTTGTAGGGGGGAGCTCTCTGCAGGAGGAGAAAATGGACGGCGGGAGGGCACGGAGCAGAGTCGGTGAGGAGCTGAGCAGGTGAAGGAGGAGGAAGCTTTCCCAGCAGGCGTGTCCTGAGTGCTTCACAGCGGAAAGTTACAGATGCTGATTTGGGAGCTGCGGCTGATTCTGGAAACAAATGGGATAAATAGTGGCATGAGAAAAAAAAAAAAAAAGTTGAAATGCCCGAAGGGCACATCTGCTGTGGCCAGCAATTCTGCAGGAAATGCACCGAAACCGGAGAGAGACGAGGAAGCTGCAGATAAACACGGCCCCCAGCGAAGCCAAACGAAGGCCACGGGCCTGAGAAGTGCCTGCAATAACGTGCTGAAGATCTGGCTTTCTTTTTTGGCTCTCTGCTGAAGTAGCTGTGTTTAACGTGGGCCCGAGACTGGCTGTACTGAATGAATTTTAATGGTGTTTTGCATTCTGAGCTCGGAACCTCTACTCCAGAGACTTTTCTGTTGCTTTCAAGGGTGTGTGTGAGAGCGCAGGGCTGCGTGGTGAGGAGGTGCACTGTAGGTCTGTTAGTCTGAAATCTGAATAATTGCACGGTCATTACATTTTGCACAAAAAAGGAATTGACTCTAAAAGCAGCTGATATGTTATCATGATTAGAGCAATAGCAATGCCCTCTTTCATCGCAGTGAAGTTTCATTGTTAGAGAGCATAATTGACATTTTTTATTGAAAGATTAAGAAGAATTTGTTTACAGTTTGCAAACAAAGAGGTTTTAATTCAACAAACTGCAGCAGGAATAAGCAGCAAGTGGCATTTTCATCTGCTGGCTGGTTAGTGCTTTAGCTGCAGAATGCTGCGGATAGAGAAGTATGAAGGAATTTGCAGCTCAATATCTGCCCAATAAAGCGTGCACCACACTGGCAAATGCCAGAAAGCTGACGGGGAGAAACCTTGAGTGCAAAGAAACAAAGGGCTATAGTTAATATTAGCACCTTTTGAGAAGACACCGCG

**2. The flanking DNA region of *Ldha* gene**

>NC_006092.5:12752120-12754120 Gallus gallus breed Red Jungle Fowl isolate RJF #256 chromosome 5, GRCg6a

CCACCACTCTCTAGCTTCAGCACTTCCCACTACTGCAATGGAAGGTAGCAGTGGAAAAATAACAGGAAGAATAAAATTCCTTATGGATACAAGTGAGGAAAATTGACCCCATCCCTACAGTCAAAGTCAGTCACTTAAAAGAAATCACAACAAATCCTGCCTCCATCTTCCATGTGCAAGAACAACGCCCTTCATTTACTCACCTCATTCTGTTGGTTTTTTTTTGTTATGAGTCTTTTCTATTAGAAGCTATCTGAAGCAGAAAATCAGTGTCAATCATGTCAGATGACTGCTCACCTCCCGAGCTTCTACTGATGCTCCTAAGATCACAAAGGACACATAAAAATTTGGCATTTGCAACTTTCTCATTTTTGTAAGCCAGGTTAGTTAACACTAGCATATTAAAACCTGTAACCCATTTTTAACAGAATCAGAAGGCTTCTGATCTGTTGACCATCACTGCAGACACGTTGGGACCATGACGGTATTGAGACATCCTAGTCAGCTCGCTCTGGCCTACTGTACTAAAGGCAGGCCCTCTGGTATTTGAATAGGCACTGGACAGCTAAGAGGAACCTGATGCTATAAGTTACCTTATGTTATAAATAATGGAGATTTGGTCTCAGTTCTGTTCTCAGTAACAAGCAGCTCAAACATGAGCAAGTGAAGATTTTACTGGATCCTATACCCCAAAGACCACACCCAGTTTTATTTGCCTGAGCCACAGAATTAGAACAAACCTACCATACCAACATTTTTATCCTAAAAACTTTGGTTTCACTGTGACAGTAGTAAAGTACCATTTGCTCTGCACTAGTTACATTTTTAATGTGCCCATTAGCCTATCCAGCCAGCCTTGTCTTAGAAGCCTCTCATCTTACATTCATAACCTTTGCAAGCCAAATTTTTTATCAAGAGAAAACACTTATCGATCTTTTTCAGTCAAATGTTCACTTTCTATCCTGCCAGACAGAGGCCAACACAGTGCTGCGTAGATGCTGGTGTCTAGAACCTGAACCGGATGAGATGTTCACGCCCATGCACACATGGCATAAATACAAATTCCTGGGATGCAGAACTAAACTTCCTAATCTGCCCTTGAGACTGCTGACGGAACAAGGTGCTCAGCCAACCCCATGTAATACAGAATGGCAGAACTACATTCTAAGGTTGCCTTTTGTTCTGCATTCATGCGGCATTGGGCCTGCTCAGCACCGGCACCTTCCCCTCTGTCTCACAGCTCCTATAGCCTCTCTGCCCCACACTGCACACCTCACAAGACTCCTTCCATCACCAGCAAGGCACAAAGCACTTCTGGTTTTTGCAACCAACATGTCCGTTGGTGGAAGGACTGGTCCTTATCATGAGAATCAAAACTGTCTATTTCTGCCTTGAGATTGATGTACATCTCTCCTTATCCTATTTTTCAAGCCTTTTCAGATGTTCCCTCAGCTGCATTTCTTGTTTCTTTGGTTTCACTAATGGACTCTCAAAGAAACGAGGCACCATCCCCGTCAGGTCACCATGACAGAACGGAATGGCCGCTGGGATGTCAGCGAGGTGTCACAGCGGCAGCAAGTTCCTTCACTTGCAAAGCAGAGCTGTGCAGATGAGTGCGAGCCTTACACACCATAAGATACACCCACGGGCACAAAAATGGCACAGGCTTGCTGAGAGCGGTTGTGTGGGACAACTCTCTACCATATTCCGGCACAGGCGGCCCAGGGAAGCTGCGGACGCGGCGCGGATCCCCTCAGACAGACTCCCACACGGAGGGCAGCGCGGCGATCACCTCAGCGCTCCCGCTGTGCCCAGAGCCCCTCAGCTCTCAGCGCGCGCCGAATAACCGTTACGGGTCGTTGTGCCGCCAGCCGCACGACCGCCCGCGCCGCTCATCCCCCCCCCCCCCGCTCCCCGCCGCCCTCCGCCCCGACGTGTCGGCGGGCACGTCCCCACCGCCGCGTCCCGCCCGCCGCACGTCAGCCGCCCGGCCCTTACTTA

**3. The flanking DNA region of *Pfkp* gene**

>NC_006089.5:11437905-11439905 Gallus gallus breed Red Jungle Fowl isolate RJF #256 chromosome 2, GRCg6a

AAATACAGTTCTCTCCTGAGAGCTGTCTTAAAGAACAATGAGTAGCTGTAATACACGCTAAAAGCAGTGAAATTGTAGGCCACTTCCTAATTAGACAAGTGGGAAAACAATTACATCAGTTATAAGGGCCAAATGCTTTATGTACCATACAACAGTTGGAAAAAAAAAAAAAAAAAAAAAAAAAAAAGTACTTTCAGAAAACATAGCTCCGTAGCACCAAAGATATACTTGGAAGTAAAAGAGACATGTCTACTTCAAGACCAAATGTAAATGGTTTAACAAAACTGAGTAAAATAAGTCAGTTCTAAAGATGAGCTAAGTGTCTGTACCAAAGAAAATCAGTGCAAAGCTTCCTCAAAGTATTGCTCAGATGAGAAATTATCAACTGCCTGTTATCTTGAAGCTGTTGTAGAAGTGTATATTAGCCTAATTCCAATTGATTTTAGAGGCAGCGTGTAAGCATTAATTTAGCTTTTCCAGAGCTACCCTTAAATAAATTGATGTGACTTCATTTCATATTGGAATCATTCAAAATGGGACTTTATAGGTGTTTATATATTTCTACAATAAGAAGTTTACCTTTGTTATTACAGTTTGTTACTTGGGTATATCTTCAAATACAGGGAAATAACACCGTAAATTCCCTACACTATCACTAGTGCAGTTACCTATACAGAAAAAAAAGTACTCAAAGTATGCAAGCTATTTGGAAATGCGTCTTTTCCATTGTCAACACACTCATCTTAGCCTCTGAGATTTGCAAATATAGAATTGCCTGCATAATAAGCATAGACATTTCTTTGCACGTACAGTTTCTGGAAGCATTTATCTCTGAGTAATATTGACTGAACTACTCAAATGACTGTACGTGTGCCTGTGACTGTGTGAAAGTTGACAGGATGAGAACGTATTTTTTGAGTAAGAATTGTTTAATGTGATGTAGTTCTTTGTTATTACAGTGTTGAATCAAAACAATAATTATTACAGCAATTAAAGTATAAAACTTGAGTCAGCATGTTGTTTCTGTTCCTGACTCTCCCTGACTGTAATTTTGGGCAAGTAGCTTAGTTCCTGATTCTGCAAATGCTTTTACTTATGTTTTACTTCATTCCACAAAGAACTGTTTCTGGAACAGCACATCAATATTTATTTAACTTAAAATAAGGGTCTGTTTCTGCTATGACCACGGGCACATCGCACAATGAAGCAGAACAAAAGGTATGGCAGAAGGACAGGGCTTCCATCCAGTGTCTCTCATTTGCTTTGCCTCTCCTCCTCCCTTAGACTCAACACGTGTCTCCAAGCTGTGAATACTTGTAGACAGGGATTTCCTTTTCCTACATGTGCCTGAATGCCTGCAGTGCTTCTAGATACTCCACACATACCGGGGGACAAAGCAGACAACGACCAGGCCCTGACATAAGCTGCGCCACACCATGGCCGAAACAACCCACTCTGCTCCTTCAGCCAGAGCTGCTTTCTTCACTAGCTGAGTCTTCTCAGCAGCAGCCAAGCGAGAAACAGAAGGTAACGCAGCACTAGAAGAACCGAGCAGTAACTGAAGCTCTAGGAACAGGCTGGGAAAGGAAGGATGATCTCTGGCAGAAGGGGATTCCCCTCGCAGAGATAAGCCCTGACAGCAGGAGGGCCCCAGAGAGAGCTCCTCACAGAAGAAGCTCCCCGGCTGCGGCTCCACACAGCACAGTGTTACTCAGCACTCCGGCGCCAGGAGGATGCGCTAACTCCGTCCGATGGAGCTCCCTCCGCCCCCCTGCCCAGACGCCAGCCATGTTCACCGCATCGCTCCCACCCCGGGCCCCGGCGGCGCGGGGGGAAGGGCAGCACGGCACCTCCCGCTCCCACCTGCAGCCCCTCCCCCGCCGCTCCCCCGCCTCGCGCACACCCCCCACCTGCCCGCTGCCTCACAAACGTAACCGCCCACCCGTGGCTCGAGACAACAGCGCGCCCTCCTATTGGCTGCTCGAGACGACCGTCAACCGCCGCTCGCGGAGGGGC

Note: The blue background is the OSN binding region.
